# Forge ahead and improve the core competitiveness of *Animal Models and Experimental Medicine* ( *AMEM* )

**DOI:** 10.1002/ame2.12307

**Published:** 2023-02-10

**Authors:** Jian‐Dong Jiang

**Affiliations:** ^1^ Academician of Chinese Academy of Engineering, Editor‐in‐Chief of Acta Pharmaceutica Sinica B, Chairman, Department of Pharmacology, Peking Union Medical College & Chinese Academy of Medical Sciences Beijing China

After 5 years of accumulation, *Animal Models and Experimental Medicine (AMEM)* has received 396 manuscripts from 46 countries and regions, including Chinese mainland, Iran, the United States, India, Israel, Nigeria, Brazil, Bangladesh, Sri Lanka, the Netherlands, Denmark, Australia, Japan, and Spain. It has become an important international exchange and display platform for innovative research results in the field of laboratory animal science and basic medicine. From 2018 to 2021, *AMEM* was indexed by CSCD, DOAJ, PubMed Central, WJCI, MEDLINE, ESCI, and other domestic and foreign scientific databases. In 2022, it was included in Elsevier's Scopus database, indicating the further improvement of *AMEM*'s international influence. In the beginning of 2023, good news kept pouring in for AMEM. First, after the first year of inclusion in the Scopus database, the first CiteScore will be obtained this year. Second, in 2023, it will receive the first Impact Factor (JIF) moving another big step forward for it to become a world‐class journal.

After changing to a bimonthly journal, *AMEM* has accelerated the construction of international frontier hotspots column. So far, seven hot topics that focus on themed issues of international basic medical research have been published, such as the study of intestinal microflora, cardiovascular and cerebrovascular disease, and neurodegenerative disease. In October 2022, the themed section on the interaction between virus and host was launched. Research papers and comments by domestic scholars on infectious diseases, such as monkeypox virus and COVID‐19 related diseases, were published on that issue. *AMEM* editorial department vigorously promoted the research results and insights of scientists from all over the world in this field online and offline. In 2023, *AMEM* will continue to make efforts to make hot columns regular ones to improve its core competitiveness and influence.

In 2021, under the leadership of Editor‐in‐chief Chuan Qin, *AMEM*'s editorial team was unprecedentedly united and active in reviewing, organizing, and contributing after the convening of the editorial board meeting, meeting the challenges of vigorous development and competition in Chinese journals in the post‐epidemic era. In the face of new opportunities and challenges, our goal is to invite manuscripts from international peers, increase the number of manuscripts of *AMEM*, maintain the quality of *AMEM*'s manuscripts, forge ahead, stand out among similar journals, and move toward becoming the world's first‐class journal!

At the beginning of the New Year, we warmly welcome more domestic and foreign experts to contribute articles to *AMEM*. We would like to express our gratitude for the support and trust of experts in laboratory animal industry and thank experts from editorial team for their hard work. *AMEM* will stay true to its original intention of its inception, continue to work hard in this field, forge ahead bravely, and go on creating brilliance. We wish everyone a happy new year!

